# IL-17 Pathway Members as Potential Biomarkers of Effective Systemic Treatment and Cardiovascular Disease in Patients with Moderate-to-Severe Psoriasis

**DOI:** 10.3390/ijms23010555

**Published:** 2022-01-05

**Authors:** Xing Wang, Hannah Kaiser, Amanda Kvist-Hansen, Benjamin D. McCauley, Lone Skov, Peter Riis Hansen, Christine Becker

**Affiliations:** 1 Department of Medicine, Division of Clinical Immunology, Icahn School of Medicine at Mount Sinai, New York, NY 10029, USA; xing.wang@mssm.edu (X.W.); benjamin.mccauley@mssm.edu (B.D.M.); 2 Department of Cardiology, University Hospital—Herlev and Gentofte, 2900 Hellerup, Denmark; lilian.hannah.kaiser@regionh.dk (H.K.); amanda.kvist-hansen@regionh.dk (A.K.-H.); Peter.Riis.Hansen@regionh.dk (P.R.H.); 3 Department of Dermatology and Allergy, University Hospital—Herlev and Gentofte, 2900 Hellerup, Denmark; lone.skov.02@regionh.dk; 4 Department of Clinical Medicine, University of Copenhagen, 2200 Copenhagen, Denmark; 5 Department of Genetics and Genomic Sciences, Icahn School of Medicine at Mount Sinai, New York, NY 10029, USA

**Keywords:** psoriasis, cardiovascular disease, Olink, proteomics, IL-17A, IL-17C, PI3/elafin, psoriasis area and severity index

## Abstract

Psoriasis is a chronic inflammatory condition associated with atherosclerotic cardiovascular disease (CVD). Systemic anti-psoriatic treatments mainly include methotrexate and biological therapies targeting TNF, IL-12/23 and IL-17A. We profiled plasma proteins from patients with moderate-to-severe psoriasis to explore potential biomarkers of effective systemic treatment and their relationship to CVD. We found that systemically well-treated patients (PASI < 3.0, *n* = 36) had lower circulating levels of IL-17 pathway proteins compared to untreated patients (PASI > 10, *n* = 23). Notably, IL-17C and PI3 were decreased with all four examined systemic treatment types. Furthermore, in patients without CVD, we observed strong correlations among IL-17C/PI3/PASI (r ≥ 0.82, *p* ≤ 1.5 × 10^−12^) pairs or between IL-17A/PASI (r = 0.72, *p* = 9.3 × 10^−8^). In patients with CVD, the IL-17A/PASI correlation was abolished (r = 0.2, *p* = 0.24) and the other correlations were decreased, e.g., IL-17C/PI3 (r = 0.61, *p* = 4.5 × 10^−5^). Patients with moderate-to-severe psoriasis and CVD had lower levels of IL-17A compared to those without CVD (normalized protein expression [NPX] 2.02 vs. 2.55, *p* = 0.013), and lower IL-17A levels (NPX < 2.3) were associated with higher incidence of CVD (OR = 24.5, *p* = 0.0028, 95% CI 2.1–1425.1). As a result, in patients with moderate-to-severe psoriasis, we propose circulating IL-17C and PI3 as potential biomarkers of effective systemic anti-psoriatic treatment, and IL-17A as potential marker of CVD.

## 1. Introduction

Examples of established systemic therapies for moderate-to-severe psoriasis include methotrexate, anti-tumor necrosis factor (TNF) agents (e.g., adalimumab, infliximab, etanercept, and certolizumab), anti-IL-12/23 antibody ustekinumab and anti-IL-23 antibodies (e.g., guselkumab, risankizumab and tildrakizumab), anti-IL-17A antibodies (e.g., secukinumab and ixekizumab), and anti-IL-17RA antibody brodalumab. Th17 cells and the IL-17 signaling pathway play a central role in psoriasis disease etiology and treatment [1,2,3]. Indeed, the IL-23, IL-17 and TNF antagonists are highly effective acting mainly through inhibition of the IL-23/IL-17 signaling pathway [2,4]. Methotrexate works through more diverse antiproliferative and immunomodulatory actions [5,6,7], and it is not known if this drug also mainly acts through the IL-17 signaling pathway. Therefore, a systematic comparison of different types of effective systemic psoriatic treatments including methotrexate would be informative and could lead to identification of biomarkers of effective treatment.

There are six known IL-17 family members including IL-17A, IL-17B, IL-17C, IL-17D, IL-17E, and IL-17F [3], with IL-17A and IL-17F being the best studied in psoriasis. Chemokines such as CCL20 and MCP-3/CCL7 are stimulated by Th17 cell cytokines, e.g., IL-17A [8,9], which may contribute to a positive IL-17 response feedback loop in psoriasis. Keratinocytes and other cells in psoriatic lesional skin may also secrete cytokines that interplay with this pathogenic network [4,10]. IL-17C is the most abundant IL-17 isoform in lesional psoriasis skin [11], shares 27% homology with IL-17A and is produced by epithelial cells rather than hematopoietic cells that produce the other IL-17 family members [10]. IL-17C shares several commonalities with IL-17A, in terms of immune and pro-inflammatory effects [1], and together with IL-17A forms a hypothetical “feed-forward” model contributing to keratinocyte hyperplasia and systemic inflammation in psoriasis [2]. Therefore, IL-17C is gaining interest as a candidate for therapeutic targeting in psoriasis and other autoimmune diseases [10].

Peptidase inhibitor 3 (PI3), also called skin-derived antileukoproteinase (SKALP) or elafin, is an elastase inhibitor first isolated from skin of patients with psoriasis [12]. Like IL-17C, PI3 is expressed predominantly by epithelial cells [13]. Expression of PI3 is increased in lesional skin and blood from patients with psoriasis and circulating levels of PI3 was shown to correlate with psoriasis disease severity [14,15,16,17,18], and is induced by IL-17A in keratinocytes [15].

Psoriasis is associated with multiple comorbidities such as cardiovascular disease (CVD), diabetes and psoriatic arthritis [19]. Increased risk of atherosclerosis includes, for example, cardiovascular mortality, stroke, myocardial infarction and peripheral artery disease [20,21,22,23,24]. Similar to psoriasis, atherosclerosis is a chronic inflammatory disease [25] and the two diseases share immunological mechanisms [26,27,28]. Along this line, systemic treatment of psoriasis with methotrexate [29] and anti-inflammatory biologics has been suggested to reduce the risk of CVD [30,31,32], although controversy remains and the role of, e.g., IL-17 in atherosclerotic CVD is unclear [3,33,34,35,36].

In this study, we explored in patients with moderate-to-severe psoriasis the plasma protein changes associated with effective systemic anti-psoriasis treatment to further inform on biological pathways and biomarkers that contribute to the disease and its treatment. Furthermore, we determined if these changes were influenced by coexistent CVD.

## 2. Results

### 2.1. Study Cohort

A total of 84 adult patients with moderate-to-severe psoriasis were included in the full study cohort. This comprised 36 systemically well-treated (Psoriasis Area and Severity Index (PASI) 0.7 [IQR 0.0–1.8] and 23 systemically untreated (PASI 13.6 [IQR 11.5–16.1]) patients (total *n* = 59, details in Materials and Methods) without significant differences in demographics (Table 1). Overall, 39 of the 84 (46.4%) patients had a history of atherosclerotic CVD including 14 (38.9%) and 10 (43.5%) in the well-treated and untreated groups, respectively. Of the 36 well-treated patients, 30 received one of four different anti-psoriatic treatments in sufficient numbers for statistical analysis, namely methotrexate (*n* = 11), adalimumab (*n* = 8), ustekinumab (*n* = 5), and secukinumab (*n* = 6). Additionally, there were other systemic treatment types with less patients treated including ixekizumab (*n* = 1), guselkumab (*n* = 1), infliximab (*n* = 3), and etanercept (*n* = 1). Characteristics of patients stratified by treatment types are reported in Table S1.

### 2.2. Effective Systemic Anti-Psoriatic Treatment Is Associated with Decreased Levels of Circulating Proteins in the IL-17 Signaling Pathway

**Figure 1:**
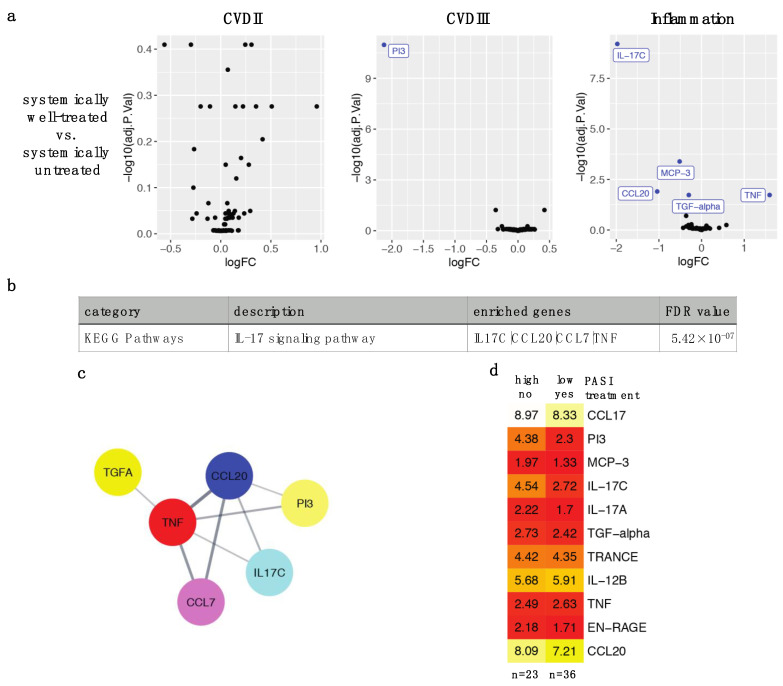
Differentially expressed proteins (DEPs) in effective systemic treatment of psoriasis. (**a**) Proteins measured by Olink CVDII, CVDIII and Inflammation panels were compared between systemically well-treated (PASI < 3.0, *n* = 36) and systemically untreated (PASI > 10.0, *n* = 23) patients with results visualized by Volcano plot. LogFC are in Log2 scale, and adjusted *p*-values < 0.05 are colored and labeled in blue. (**b**) Gene set enrichment was performed using STRING enrichment plugin against Gene Ontology, KEGG and Reactome pathway databases. Enrichment of IL-17 signaling pathway was detected, which contained IL-17C, CCL20, TNF and MCP-3/CCL7. (**c**) Protein-protein interaction network analysis of DEPs was performed and visualized by Cytoscape with STRING plugin. (**d**) Heatmap showing median NPX values of DEPs between PASI > 10.0 no treatment (*n* = 23) and PASI < 3.0 with treatment groups (*n* = 36).

**Figure 2:**
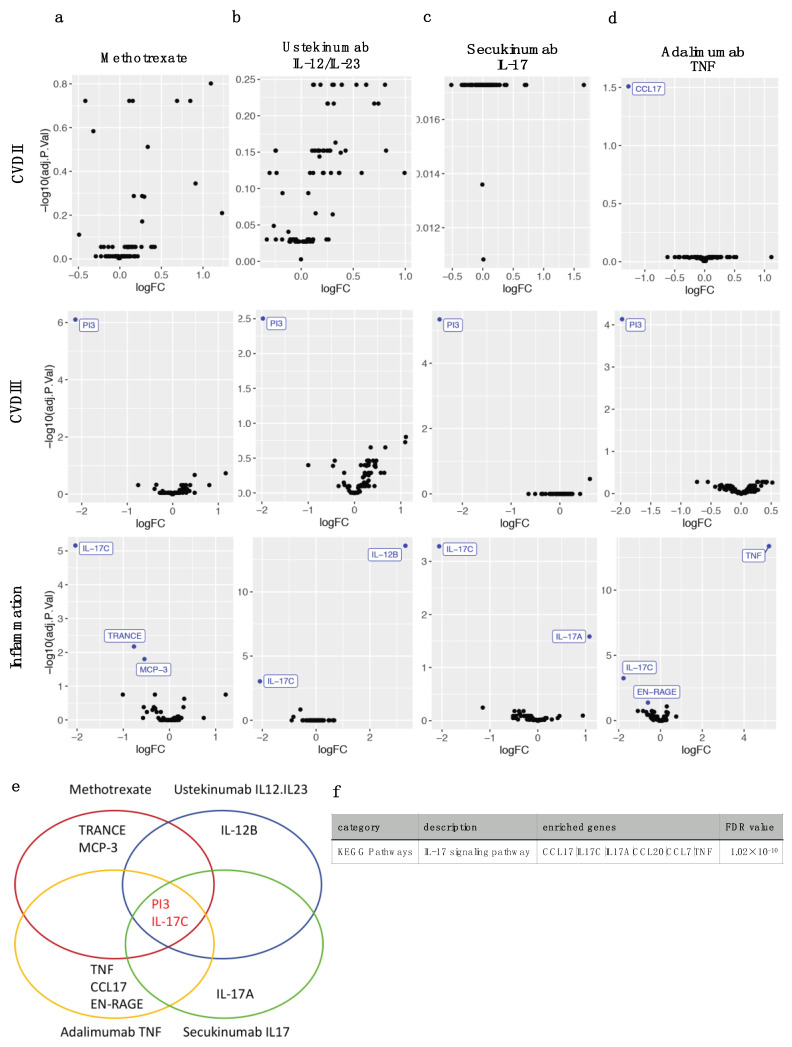
Differentially expressed proteins (DEPs) between various types of systemic treatment of psoriasis and no treatment. Normalized protein expressions of Olink CVDII, CVDIII and Inflammation panels were compared separately between various types of effective antipsoriatic treatment (PASI < 3.0) and no treatment (PASI > 10.0, *n* = 23), and visualized by Volcano plots. (**a**) Methotrexate (*n* = 11); (**b**) Ustekinumab (IL-12/23 antibody, *n* = 5); (**c**) Secukinumab (IL-17 antibody, *n* = 6); (**d**) Adalimumab (TNF antibody, *n* = 8). LogFC are in Log2 scale, and adjusted *p*-values < 0.05 are colored and labeled in blue. (**e**) Venn diagram of DEPs resulting from various types of treatment show shared PI3 and IL-17C protein changes. (**f**) Gene set enrichment for all 11 DEPs from each of the treatment types identified enrichment of the IL-17 signaling pathway.

To explore the effects of effective anti-psoriatic treatment on plasma proteins, we compared plasma proteins from 36 systemically well-treated versus 23 systemically untreated patients. Differentially expressed proteins (DEPs) after adjusting for multiple testing were PI3, IL-17C, MCP-3/CCL7, CCL20, TGF-α and TNF-α/TNF (FDR < 0.05), the first five proteins were decreased, while TNF was increased with treatment (Figure 1a). Protein-protein interaction network analysis and pathway analysis identified that these proteins were connected and highly enriched in the IL-17 signaling pathway (*p* = 5.42 × 10^−7^) (Figure 1b,c). A heatmap of above DEPs and DEPs from individual treatment types (Figure 2) illustrated that PI3 (normalized protein expression [NPX] 4.38 vs. 2.3) and IL-17C (4.54 vs. 2.72) had the greatest decrease in expression in well-treated compared to untreated patients. MCP-3/CCL7 (1.97 vs. 1.33), CCL20 (8.09 vs. 7.21) and TGF-α (2.73 vs. 2.42) also showed decreased expression in well-treated patients but to a lesser degree than PI3 and IL-17C (Figure 1d).

### 2.3. Four Different Types of Systemic Anti-Psoriatic Treatment Share Association with Lower Levels of PI3 and IL-17C

Among the 36 systemically well-treated patients, 30 exclusively used either methotrexate (*n* = 11), ustekinumab (*n* = 5), secukinumab (*n* = 6) or adalimumab (*n* = 8) with sufficient numbers available for statistical analysis. NPXs in each of these 4 treatment groups were compared separately with the untreated group (*n* = 23). Methotrexate was associated with lower PI3, IL-17C, MCP-3/CCL7 and TRANCE (Figure 2a), ustekinumab with lower PI3, IL-17C, and higher IL-12B (Figure 2b), secukinumab with lower PI3, IL-17C and higher IL-17A (Figure 2c), and adalimumab with lower PI3, IL-17C, CCL17, EN-RAGE, and higher TNF levels (Figure 2d), respectively. Accordingly, all 4 systemic anti-psoriatic treatments were linked with lower PI3 and IL-17C (Figure 2e). Functional enrichment by STRING identified that 6 of 11 DEPs were members of the IL-17 signaling pathway (FDR = 1.02 × 10^−10^) including IL-17A, IL-17C, CCL20, MCP-3/CCL7, TNF and CCL17 (Figure 2f).

### 2.4. The Identified IL-17 Pathway Proteins Strongly Correlate with Each Other and with PASI

**Figure 3:**
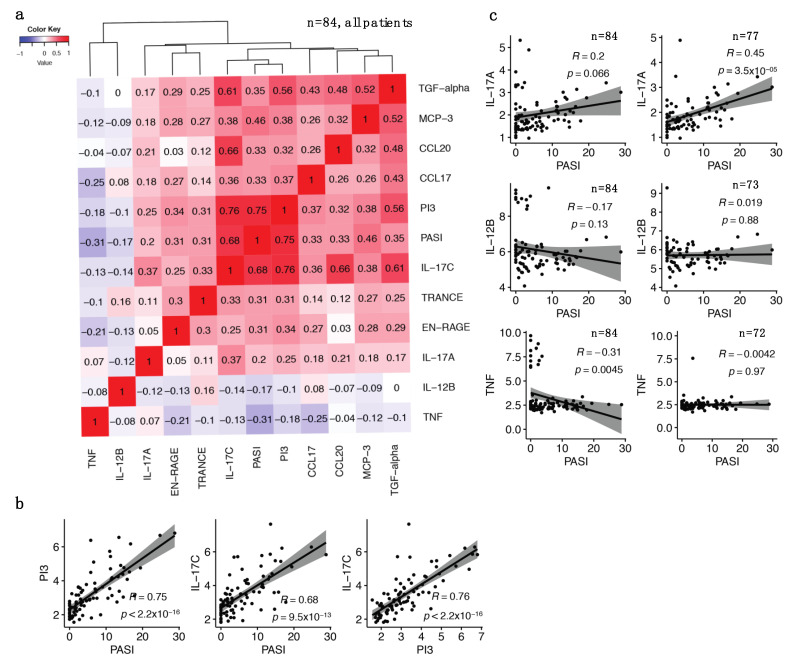
Correlation matrix of selected differentially expressed proteins (DEPs) and PASI for all patients (*n* = 84). (**a**) Correlation matrix heatmap shows correlations between selected DEPs and PASI for all patients (*n* = 84); (**b**) Pearson correlation scatter plots between selected DEPs proteins and PASI for all patients (*n* = 84), and correlation between PI3 and IL-17C. Pearson correlation coefficients R are shown with associated *p*-values, and y-axis shows protein NPX values. (**c**) Pearson correlation scatter plots before (*n* = 84, left panel) and after (*n* = 77, *n* = 73, and *n* = 72, respectively, right panel) excluding the patients with paradoxically elevated levels of targeted cytokines receiving anti-IL-17A (*n* = 7), anti-IL-12/23 (*n* = 11), and anti-TNF (*n* = 12), respectively, from the analyses.

Using the entire patient cohort (*n* = 84), correlations between DEPs described in Figure 2 and PASI along with inter-correlations between these proteins were assessed. PI3, IL-17C and PASI clustered tightly together (Figure 3a) with correlation coefficients of 0.75 (*p* < 2.2 × 10^−16^) for the PI3/PASI pair and 0.68 (*p* = 9.5 × 10^−13^) for the IL-17C/PASI pair (Figure 3b). PI3 and IL-17C were also highly correlated with each other (r = 0.76, *p* < 2.2 × 10^−16^) (Figure 3b). Other significant correlations with PASI included the IL-17 pathway proteins MCP-3/CCL7 (r = 0.46, *p* = 1.1 × 10^−5^), CCL20 (r = 0.33, *p* = 0.0021) and CCL17 (r = 0.33, *p* = 0.0022), as well as TGF-α (r = 0.35, *p* = 0.00095), TRANCE (*p* = 0.31, *p* = 0.0036) and EN-RAGE (r = 0.31, *p* = 0.0045) (Figure S2).

In the full study cohort (*n* = 84), we did not detect correlation between IL-17A and PASI (r = 0.2, *p* = 0.066) (Figure 3c, upper left). However, several patients had very high levels of IL-17A despite receiving anti-IL-17A agents (secukinumab or ixekizumab) possibly because both active and antibody-bound IL-17A were detected by the Olink assay. After removing patients receiving these two agents (*n* = 7) we observed a significant IL-17A/PASI correlation (r = 0.45, *p* = 3.5 × 10^−5^) (*n* = 77, Figure 3c, upper right). We observed a similar phenomenon with apparent elevated levels of the targeted cytokines in patients receiving anti-IL-12/23 (ustekinumab) or anti-IL-23 (guselkumab) therapies (Figure 3c, middle left) or anti-TNF agents (Figure 3c, lower left), respectively. When patients receiving IL-12/23 therapies were excluded from the analysis, the IL-12B/PASI correlation (*n* = 73) remained nonsignificant (Figure 3c, middle right). The significant TNF/PASI correlation in the entire cohort (*n* = 84) (r = −0.31, *p* = 0.0045) disappeared (*n* = 72, r = −0.0042, *p* = 0.97, Figure 3c, lower right panel) when patients that received TNF antagonists (adalimumab, infliximab or etanercept) were removed.

### 2.5. Increased Correlations between IL-17 Pathway Proteins and PASI in Patients without CVD Are Weakened with CVD

**Figure 4:**
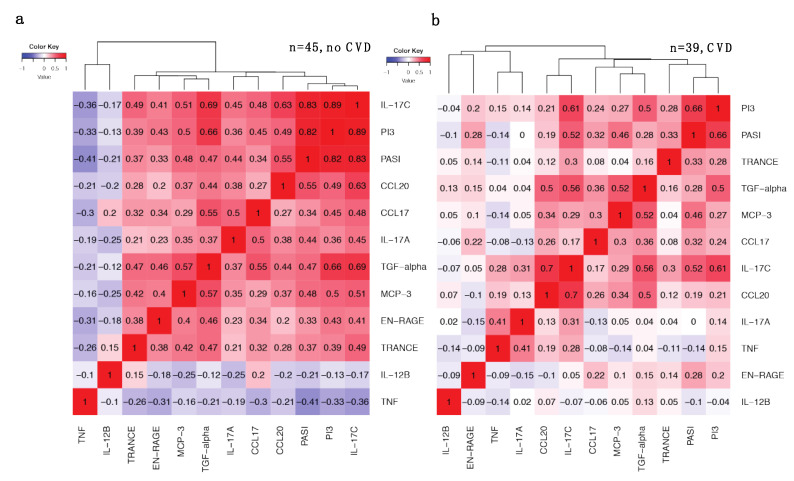

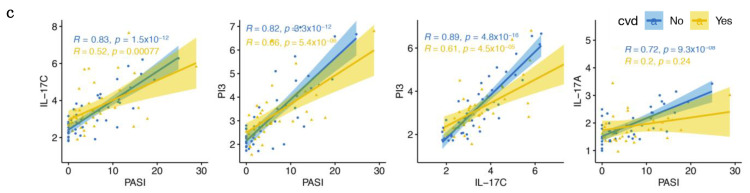
Correlations of selected differentially expressed proteins (DEPs) and PASI for patients with (*n* = 39) or without CVD (*n* = 45). (**a**) Correlation matrix heatmap shows correlations between selected DEPs and PASI for patients without CVD (*n* = 45); (**b**) Correlation matrix heatmap shows correlations between selected DEPs and PASI for patients with CVD (*n* = 39); (**c**) Pearson correlation scatter plots between selected DEPs and PASI for patients with psoriasis and CVD or without CVD. Pearson correlation coefficients R are shown with associated *p*-values, and y-axis shows protein NPX values.

We next stratified the patient cohort into two groups to test the effect of CVD on the DEPs described above and noted that CVD status had a profound effect on many of these correlations (Figure 4). Indeed, we observed increased correlations between PI3, IL-17C and PASI in the patient group without CVD (*n* = 45, Figure 4a), where IL-17C/PI3 correlation increased to r = 0.89 (*p* = 4.8 × 10^−16^, Figure 4a,c), and decreased in the presence of CVD (r = 0.61, *p* = 4.5 × 10^−5^). The IL-17C/PASI correlation also increased to r = 0.83 (*p* = 1.5 × 10^−12^) and PI3/PASI increased to r = 0.82 (*p* = 3.3 × 10^−12^) in patients without CVD (Figure 4a,c). In the CVD group (*n* = 39), these correlations were considerably weaker (Figure 4b,c) where the correlation coefficients of IL-17C/PASI and PI3/PASI decreased to 0.52 (*p* = 0.00077) and 0.66 (*p* = 5.4 × 10^−6^), respectively. A decrease in correlation with PASI was also observed for other DEPs in the presence of CVD (Figure S3). For example, CCL20/PASI decreased from r = 0.55 (*p* = 9.6 × 10^−5^) to r = 0.19 (*p* = 0.25) and TGF-α from r = 0.47 (*p* = 0.0013) to r = 0.28 (*p* = 0.089).

The IL-17A/PASI correlation was r = 0.45 (*p* = 3.5 × 10^−5^) in the entire cohort after excluding patients receiving anti-IL-17A antibodies (Figure 3c, *n* = 77). However, after stratifying patients by CVD status, we observed increased correlation (r = 0.72, *p* = 9.3 × 10^−8^) in patients without CVD (Figure 4c and Figure S3b, *n* = 41), which was lost in patients with CVD (r = 0.2, *p* = 0.24, Figure 4c and Figure S3d, *n* = 36).

The DEPs with decreased correlation with PASI in the presence of CVD were highly enriched in the IL-17 signaling pathway including IL-17C, PI3, IL-17A and CCL20.

### 2.6. IL-17A Levels Are Lower in Patients with Moderate-to-Severe Psoriasis and CVD Compared to Patients without CVD

**Figure 5:**
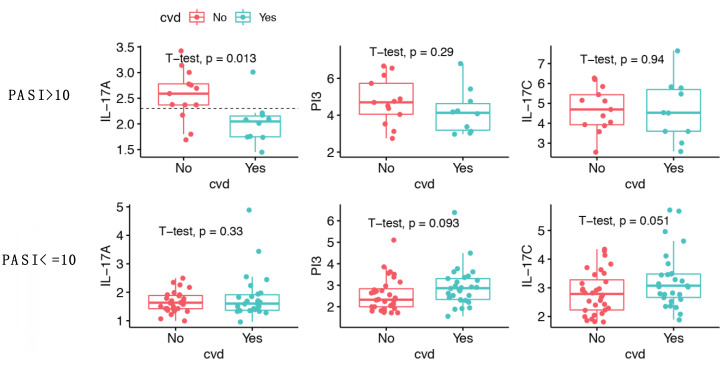
Comparison of IL-17A, IL-17C and PI3 protein levels in patients stratified by CVD and psoriasis severity. Boxplot comparison of IL-17A, IL-17C and PI3 protein levels in patients with CVD (*n* = 39) and without CVD (*n* = 45), y-axis shows protein NPX values. A t-test was carried out and resulting *p*-values are shown. In patients with PASI > 10 the mean normalized protein expression (NPX) of IL-17A was 2.55 in subjects without CVD, compared to 2.02 in those with CVD (*p* = 0.013). Fisher’s exact test showed that an IL-17A NPX value below 2.3 was associated with higher incidence of CVD for patients with PASI > 10 (horizontal dotted line). In patients with PASI ≤ 10, the mean IL-17C NPX was 2.82 in patients without CVD compared to 3.27 in patients with CVD (*p* = 0.051).

Examination of the correlation scatter plots suggested that the loss of IL-17A/PASI correlation in patients with CVD might be due to lower IL-17A levels in patients with higher PASI (Figure 4c, right). Therefore, we further stratified the 84 patients into two groups based on PASI > 10 or ≤10, and compared levels of IL-17A, IL-17C and PI3 between patients with and without CVD (Figure 5). In patients with PASI > 10, IL-17A levels were significantly lower in individuals with CVD (Figure 5, upper left) compared to those without CVD (NPX 2.02 vs. 2.55, *p* = 0.013). Most patients with moderate-to-severe psoriasis (PASI > 10) and CVD had IL-17A levels below 2.3 (9 vs. 1 patient), whilst patients with moderate-to-severe psoriasis but no CVD had IL-17A levels above 2.3 (10 vs. 3 patients), and an IL-17A level below 2.3 was associated with increased incidence of CVD (odds ratio 24.46, *p* = 0.0028, 95% confidence interval 2.1–1425.1). In contrast, a difference in IL-17A levels was not seen in patients with PASI ≤ 10 (Figure 5, lower left, *p* = 0.33). Additionally, we did not detect a difference in IL-17C and PI3 levels between patients with or without CVD after stratification for PASI > 10 or ≤10 (Figure 5, middle and right). However, we observed borderline significant higher IL-17C levels in patients with CVD when PASI ≤ 10 (3.27 vs. 2.82, *p* = 0.051, Figure 5, lower right).

## 3. Discussion

We used Olink proteomics and computational analyses to characterize circulating protein biomarkers associated with effective systemic treatment of psoriasis and their relationship with CVD. Network and pathway analyses identified that the DEPs were connected and highly enriched in the IL-17 signaling pathway reinforcing the central role of this pathway in psoriasis pathology and treatment. Indeed, previous studies have shown that in clinical responders, treatment with methotrexate reduces IL-17A mRNA [37], adalimumab reduces IL-17C mRNA [38], secukinumab reduces IL-17A and IL-17C mRNA in psoriatic lesions [39], and ustekinumab reduces blood levels of IL-17A [40]. In this study we confirm and extend these findings by demonstrating that effective treatment with each of the four different systemic anti-psoriatic agents was associated with concomitantly reduced circulating levels of IL-17C and PI3 indicating that IL-17C and PI3 have potential as blood biomarkers of effective systemic treatment.

IL-17C and PI3 proteins are also interesting targets that merit further investigation in psoriasis treatment. Recombinant or overexpressed PI3 in animal models has demonstrated a protective role in a wide range of experimental tissue injuries including, for example, viral myocarditis, myocardial infarction, heart transplantation, colitis, pulmonary inflammation and vein graft degeneration [41,42,43,44,45,46]. An investigational IL-17C-specific antibody (MOR106) showed promising results in experimental studies for treatment of psoriasis and atopic dermatitis [47,48], however, phase II clinical trials for its use in atopic dermatitis were halted due to lack of efficacy (ClinicalTrials.gov Identifier NCT03568071). Efforts to examine clinical effects of this drug in psoriasis have not yet been reported and appear worthy of further exploration.

IL-17C and PI3 levels were highly correlated to each other and PASI. Study of the relationship between IL-17C and PI3 is limited. IL-17C is induced in keratinocytes by bacterial and inflammatory stimuli, regulates epithelium innate immune responses through an autocrine loop [49], and is critical for mediating neutrophil migration and amplification of epithelial inflammation [47]. While neutrophil elastase is necessary for killing of intracellular pathogens upon phagocytosis [50], PI3 quickly antagonizes elastase activity and contributes to resolution of inflammation, maintaining tissue integrity [42,51]. Therefore, the balance of IL-17C, neutrophil elastase and PI3 may be important for restoration and maintenance of host tissue integrity in the face of endogenous and exogeneous irritants and disrupting this balance may contribute to chronic inflammation leading to various diseases including atherosclerosis and CVD [13,52,53].

Several patients receiving anti-psoriatic biologics had high levels of the respective targeted proteins. This may be due to the measurement of both the active and antibody-bound inactive forms of the protein by the assay, which has occasionally been reported for other assays [54]. It has also been shown that Oink TNF assays can measure both the free form of TNF and TNF in complex with infliximab or adalimumab (personal communication with Olink). Furthermore, when plasma IL-17A proteins are inhibited by the antagonistic antibody, the IL-17A positive feedback loop [55] may exert a compensatory effect and result in much higher levels of combined active and antibody-bound inactive IL-17A. Excluding the patients receiving anti-IL-17A therapies from analysis enabled us to detect that the IL-17A and PASI correlation was dependent on CVD status. The loss of IL-17A/PASI correlation in the presence of atherosclerotic CVD, together with the decreased correlations of PI3, IL-17C, CCL20 and TGF-α with PASI in these patients, clearly merits further investigation and may contribute to further understanding of the links between psoriasis and CVD. Similarly, we observed that patients receiving TNF and IL-12/IL-23 antagonistic therapies showed higher rather than lower levels of TNF or IL-12B proteins, respectively, whereas IL-23 was not included in the Olink panels. This potential discrepancy in measuring levels of targeted proteins in patients receiving biologics needs to be considered in future studies.

IL-17A levels were significantly lower in patients with moderate-to-severe psoriasis and CVD than in those without CVD. The analysis showed that lower plasma IL-17A levels were associated with increased incidence of CVD in these patients and therefore IL-17A may serve as a CVD biomarker. There is ample evidence that IL-17 has roles in CVD although there are uncertain aspects determined by its dual proatherogenic and antiatherogenic actions [35,36]. For example, in patients with acute myocardial infarction low serum IL-17A levels have been linked with mortality and recurrent major cardiovascular events, suggesting a protective role for IL-17A [56]. Additionally, a recent meta-analysis demonstrated that treating psoriasis with anti-IL-17A secukinumab did not show beneficial effect on CVD risk imaging biomarkers including aortic vascular inflammation or flow-mediated dilatation [57]. Conversely, studies have suggested that anti-IL-17A treatment may reduce risk of CVD in patients with psoriasis albeit that adequately powered randomized trials with clinical endpoints are awaited [3,30]. Our results should be interpreted in the light of limitations including a relative low number of study subjects of Caucasian descent and the data-driven computational analyses that preclude relevant a priori sample size calculations. We conclude that in patients with moderate-to-severe psoriasis, circulating levels of IL-17C and PI3 may serve as biomarkers of effective systemic anti-psoriatic treatment and that concurrent CVD is linked with modulation of the relationship between IL-17 pathway proteins and psoriasis disease activity.

## 4. Materials and Methods

### 4.1. Patient Recruitment

This study was conducted as part of a large multi-scale study investigating the association between CVD and psoriasis (Ethical Committee ID H-17003458) [58]. A total of 86 Danish adult patients with moderate-to-severe psoriasis were recruited. Moderate-to-severe psoriasis was defined as a PASI > 10 [4,19,59,60] at inclusion or as receiving systemic anti-psoriatic treatment. In total, 84 patients were included in the analysis after performing data quality control steps. Approximately half of the cohort received systemic anti-psoriatic treatment (*n* = 44). Additionally, almost half of the entire cohort had prior atherosclerotic CVD events (*n* = 39 including peripheral artery disease, myocardial infarction, stroke and/or coronary revascularization) at least 6 months before inclusion, and are defined as patients with CVD in this study. All patients were interviewed and clinically examined with PASI determined for each patient. The criteria for systemically well-treated patients was PASI < 3 (*n* = 36) and no changes in systemic anti-psoriatic therapy within the last 3 months [61,62]. Systemically untreated patients (*n* = 23) were classified as patients with moderate-to-severe psoriasis with PASI > 10 and without biologic therapy within 5 half-lives of plasma elimination for the specific drug and/or no other systemic anti-psoriatic therapy within 1 month before inclusion. The terms ‘systemically well-treated’ and ‘systemically untreated’ are used throughout the text for effective systemic treatment and no systemic treatment, respectively. A total of 25 patients with PASI between 3 and 10 (*n* = 25) were not included in the initial comparison.

### 4.2. Sample Collection and Plasma Protein Quantification

Blood samples were collected in ethylenediaminetetraacetic acid (EDTA) tubes, centrifuged for 10 min at 2000 rpm to recover plasma and then stored at −80 °C. Aliquots of stored plasma samples were measured using the CVDII, CVDIII and Inflammation panels (Supplementary Figure S1) of OLINK Proseek^®^ Multiplex proximity extension assay (Olink Bioscience, Uppsala, Sweden) [63,64]. A total of 266 unique proteins were measured and reported as normalized protein expression (NPX) in Log2 scale.

### 4.3. Statistical Analyses

Data analysis was carried out within RStudio environment using R version 3.6.1. Continuous demographic variables are presented as mean ± SD and median (IQR), and categorical variables are presented as a number with percentages. Normality of data distribution was assessed by QQ-plots and histograms. Data were analyzed by χ^2^ test for categorical variables, Student t-test for parametric continuous variables, and Mann–Whitney Wilcoxon test for non-parametric continuous variables, respectively.

Olink NPX values reported in Log2 scale underwent quality control review. Twenty proteins measured below the Limit of Detection (LOD) in more than 40% of samples were filtered out (Figure S1). Protein changes in CVDII, CVDIII and Inflammation panels were analyzed separately. Seven proteins were measured by both CVDII and Inflammation panels, and three were measured by both CVDIII and Inflammation panels. The correlation coefficient of these repeated measurements ranged from 0.88 to 0.99. Density plots and box plots were used to review data distribution before and after protein filtering. Multidimensional scaling plots (MDS) were used to examine the similarities and dissimilarities between samples in an unsupervised manner, and outliers were excluded from downstream analysis. DEPs were analyzed following the limma-trend framework from the limma package (Version 3.42.2) [65]. *p*-values of DEPs were adjusted for multiple testing using the Benjamini–Hochberg method, which controls the false discovery rate (FDR). FDR < 0.05 was used as the cutoff to define DEPs. Fold change and FDR adjusted *p*-values are shown in Volcano plots drawn using R packages ggplot2 and ggrepel. Median rather than mean values were used to illustrate protein levels in the heatmap comparison due to very high levels of IL-12B, TNF or IL-17A in several patients receiving these respective targeting therapies (see Section 3). Correlation matrices were calculated for Pearson’s correlation coefficients and then visualized by heatmap using heatmap.2 function from the gplots package. Box plots and scatter plots were drawn using the ggboxplot and ggscatter functions from the ggpubr package. Fisher’s exact test was used to examine the association between IL-17A levels and CVD. Protein-protein interaction network analysis was performed with Cytoscape (Version 3.7.1) [66] StringAPP plugin [67] and functional enrichment with STRING enrichment plugin against datasets including KEGG [68], Reactome [69] and Gene Ontology [70] databases.

## Figures and Tables

**Table 1:** Baseline characteristics of study population.

	Entire Population (*n* = 84)	Systemically Well-Treated (*n* = 36)	Systemically Untreated (*n* = 23)	*p*-Value
Age, years	59.0 ± 10.9	59.9 ± 8.9	58.7 ± 13.0	0.685
Sex, male, *n* (%)	61 (72.6)	24 (66.7)	20 (87.0)	0.081
PASI	3.6 (1.2–11.0)	0.7 (0.0–1.8)	13.6 (11.5–16.1)	<0.001
BMI (kg/m^2^)	30.0 ± 5.6	30.4 ± 5.6	30.4 ± 5.6	0.981
Atherothrombotic CVD, *n* (%)	39 (46.4)	14 (38.9)	10 (43.5)	0.726
Medically treated diabetes, *n* (%)	21 (25.0)	12 (33.3)	6 (26.1)	0.555
Statin therapy, *n* (%)	41 (48.8)	19 (52.8)	12 (52.2)	0.964
PsA verified by rheumatologist, *n* (%)	21 (25.0)	12 (33.3)	3 (13.0)	0.081

Data are reported as mean ± SD or median (IQR) for continuous variables. *p*-values are calculated between systemically well-treated and systemically untreated patients (total, *n* = 59). PASI, psoriasis area and severity index; BMI, body mass index; CVD, cardiovascular disease; PsA, psoriatic arthritis.

## Data Availability

Please contact the corresponding author to discuss availability of the Olink data presented in this study.

## Supplementary Materials

The following supporting information can be downloaded at: https://www.mdpi.com/article/10.3390/ijms23010555/s1.
