# Supplementary material for: IL-17 Pathway Members as Potential Biomarkers of Effective Systemic Treatment and Cardiovascular Disease in Patients with Moderate-to-Severe Psoriasis
Source: Int J Mol Sci. 2022 Jan 5;23(1):555. doi: 10.3390/ijms23010555 (PMC8745093; doi:10.3390/ijms23010555)
Supplement: Supplementary file 1 [file ijms-23-00555-s001.zip › ijms-1522273-supplementary.pdf]

Supplementary Table S1. Characteristics of patients (n=30) grouped by type of systemic anti-psoriatic treatment.

	**Methotrexate (n=11)**	**Adalimumab (n=8)**	**Secukinumab (n=6)**	**Ustekinumab (n=5)**
Age, years	61.0 (58.5-64.0)	60.5 (57.5-63.3)	59.5 (55.0-70.0)	64.0 (58.0-66.0)
Sex, male, n (%)	6 (54.5)	6 (75.0)	3 (50.0)	3 (60.0)
PASI	1.2 (0.3-1.8)	0.9 (0.0-2.0)	0 (0.0-0.9)	0 (0.0-0.6)
BMI (kg/m^2)	31.4 (28.6-32.7)	34.9 (29.6-38.3)	29.5 (27.9-35.5)	30.0 (27.9-30.8)
Medically treated diabetes, n (%)	6 (54.5)	4 (50.0)	1 (16.7)	1 (20.0)
PsA verified by rheumatologist, n (%)	5 (45.5)	2 (25.0)	4 (66.7)	0 (0)

Data are reported as median (IQR) for continuous variables. PASI, psoriasis area and severity index; BMI, body mass index; PsA, psoriatic arthritis.

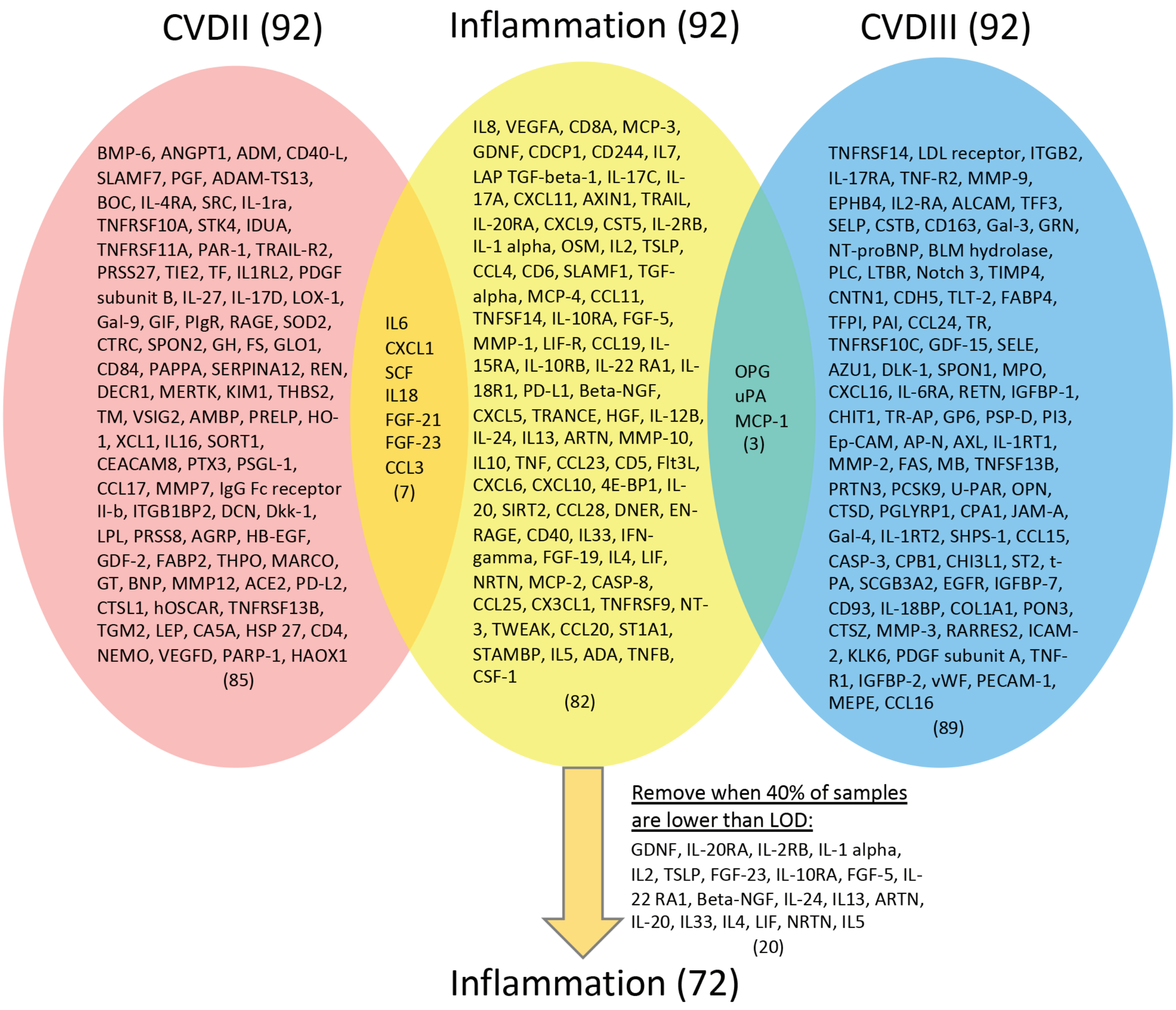

Supplementary Figure S1. Olink CVDII, CVDIII and Inflammation protein panels.

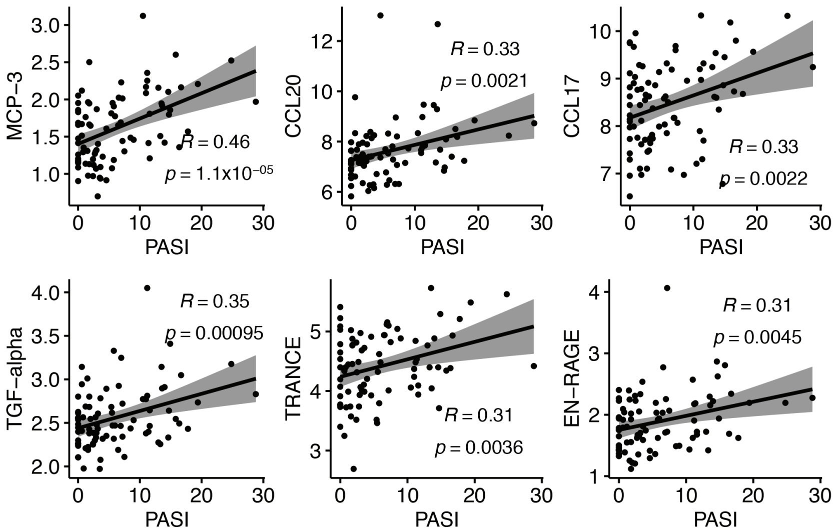

Supplementary Figure S2. Pearson correlation scatter plots between selected differentially expressed proteins and PASI for all patients (n=84).

Pearson correlation coefficients R are shown with associated p-values, and y-axis shows protein NPX values.

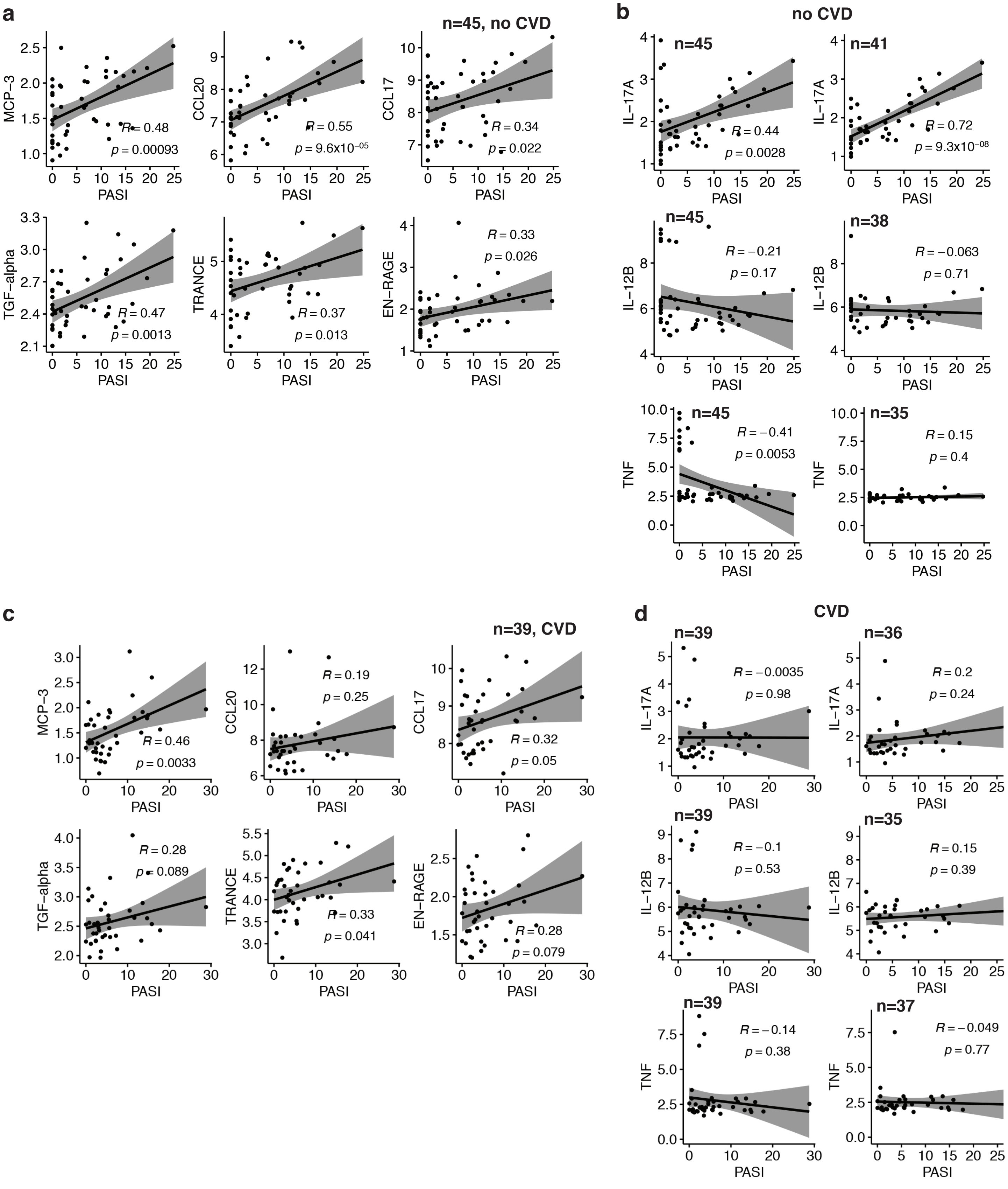

Supplementary Figure S3. Pearson correlation scatter plots between selected differentially expressed proteins and PASI.
Pearson correlation coefficients R are shown with associated p-values, and y-axis shows protein NPX values for **a.** Patients without CVD (n=45). **b.** Patients without CVD (n=45, left panel) compared to adjusted groups after exclusion of patients receiving anti-IL-17A (n=4), anti-IL-12/23 (n=7) and anti-TNF (n=10), respectively (right panel). **c.** Patients with CVD (n=39), **d.** Patients with CVD (n=39, left panel) compared to adjusted groups after exclusion of patients receiving anti-IL-17A (n=3), anti-IL-12/23 (n=4) and anti-TNF (n=2), respectively (right panel).
